# Seasonal and Weekly Patterns of Korean Adolescents’ Web Search Activity on Insomnia: Retrospective Study

**DOI:** 10.2196/52977

**Published:** 2024-10-11

**Authors:** Kwangyeol Baek, Jake Jeong, Hyun-Woo Kim, Dong-Hyeon Shin, Jiyoung Kim, Gha-Hyun Lee, Jae Wook Cho

**Affiliations:** 1 School of Biomedical Convergence Engineering Pusan National University Busan Republic of Korea; 2 Center for Artificial Intelligence Research Pusan National University Busan Republic of Korea; 3 Department of Information Convergence Engineering Pusan National University Busan Republic of Korea; 4 Department of Neurology Pusan National University Yangsan Hospital Pusan National University School of Medicine Yangsan Republic of Korea; 5 Department of Neurology Pusan National University Hospital Pusan National University School of Medicine Busan Republic of Korea; 6 Sleep Tech Research Center Bitsensing Inc Seongnam Republic of Korea

**Keywords:** insomnia, sleep, internet search, adolescents, school, seasonal, weekly, NAVER, infodemiology, inforveillance

## Abstract

**Background:**

Sleep deprivation in adolescents is a common but serious public health issue. Adolescents often have a progressive circadian delay and suffer from insufficient sleep during weekdays due to the school schedule. Temporal patterns in internet search activity data can provide relevant information for understanding the characteristic sleep problems of the adolescent population.

**Objective:**

We aimed to reveal whether adolescents exhibit distinct temporal seasonal and weekly patterns in internet search activity on insomnia compared to adults.

**Methods:**

We hypothesized that adolescents exhibit larger variations in the internet search volume for insomnia, particularly in association with the school schedule (e.g., academic vacations and weekends). We extracted the daily search volume for insomnia in South Korean adolescents (13-18 years old), adults (19-59 years old), and young adults (19-24 years old) during the years 2016-2019 using NAVER DataLab, the most popular search engine in South Korea. The daily search volume data for each group were normalized with the annual median of each group. The time series of the search volume was decomposed into slow fluctuation (over a year) and fast fluctuation (within a week) using fast Fourier transform. Next, we compared the normalized search volume across months in a year (slow fluctuation) and days in a week (fast fluctuation).

**Results:**

In the annual trend, 2-way ANOVA revealed a significant (group) × (month) interaction (*P*<.001). Adolescents exhibited much greater seasonal variations across a year than the adult population (coefficient of variation=0.483 for adolescents vs 0.131 for adults). The search volume for insomnia in adolescents was notably higher in January, February, and August, which are academic vacation periods in South Korea (*P*<.001). In the weekly pattern, 2-way ANOVA revealed a significant (group) × (day) interaction (*P*<.001). Adolescents showed a considerably increased search volume on Sunday and Monday (*P*<.001) compared to adults. In contrast, young adults demonstrated seasonal and weekly patterns similar to adults.

**Conclusions:**

Adolescents demonstrate distinctive seasonal and weekly patterns in internet searches on insomnia (ie, increased search in vacation months and weekend–weekday transitions), which are closely associated with the school schedule. Adolescents’ sleep concerns might be potentially affected by the disrupted daily routine and the delayed sleep phase during vacations and weekends. As we demonstrated, comparing various age groups in infodemiology and infoveillance data might be helpful in identifying distinctive features in vulnerable age groups.

## Introduction

Getting enough sleep is crucial in maintaining overall health, particularly during adolescence, when sleep plays a vital role in physical development and emotional stability [1]. A comprehensive review by Tarokh et al [2] emphasized the importance of sufficient sleep for adolescents’ cognitive functioning and mental health. Research has shown that insufficient sleep can impair cognitive functioning, academic performance, and mental health, with sleep-deprived adolescents being more prone to depression, anxiety, and behavioral problems [3]. According to the National Sleep Foundation of the United States, sufficient sleep for people aged 14-17 years is about 8-10 hours, and at least 7 hours of sleep is highly recommended [4]. However, many adolescents suffer from insufficient sleep, particularly during weekdays. The average weekday sleep time for adolescents was estimated as 7.46 hours in North American samples and 7.64 hours in Asian samples [5]. In the United States, 57.8% of middle school and 72.7% of high school students report insufficient sleep duration (less than 8 hours) [6]. In “Health Behavior School-aged Children” survey data in Canada and 23 European countries, 13.7%-68.0% of adolescents do not meet sleep recommendations on school days [7]. In another study in South Korea, 84.3% of high school students reported weekday sleep of less than 7 hours [8]. Adolescents, particularly those in the later stages (typically aged 13-18 years), often require more sleep than prepubertal children (generally under 10 years old) [8-10]. Despite having a progressive circadian delay and a reduced homeostatic sleep drive, adolescents still have to wake up early for school start times, which contributes to their sleep deficiency [10,11]. Psychosocially, they are exposed to various cultural and social issues, such as academic stress, the circle of friends, and online media.

Therefore, of many sleep disorders, insomnia (especially difficulty in initiating sleep) is the most prevalent symptom among adolescents, along with daytime sleepiness [5]. This trend is a worldwide phenomenon. Research on interviews in 4 European countries revealed that 25% of teenagers aged 15-18 years have insomnia symptoms and 4% of them meet the diagnostic criteria for insomnia from the *Diagnostic and Statistical Manual of Mental Disorders, Fourth Edition* (DSM-IV) [12]. Another study showed that 14.9% of Chinese teenagers aged 12-18 years have difficulty falling asleep at least 4 days a month [13]. In addition, a survey of about 26,000 middle and high school students in South Korea found that 27.4% of adolescents had insomnia, with the rate increasing with age [14]. The prevalence rate of insomnia is as high as that of depression in adolescents, but diagnosis and treatment are not adequately performed in adolescents [15].

Searching on the internet for medical knowledge, diseases, and health information has become increasingly common in recent years. According to a systematic review by Daraz et al [16], up to 80% of internet users seek health information online, with search engines being the most common starting point for health queries. Additionally, a previous study found that 73.4% of respondents used the internet as their source for antibiotic-related information [17]. Information about internet search patterns is helpful in analyzing specific characteristics of human behavior [18]. Recently, many epidemiology studies have been published using online search data from social media platforms, such as Google, Facebook, and Twitter [19-21]. Reflecting on this trend, Eysenbach [22] proposed 2 major concepts: *infodemiology*, which studies epidemiology related to public health and policy through electronic media, such as the internet, and *infoveillance*, which monitors a longitudinal trace of informational epidemiology metric for observation and tendency analysis.

Recently, a few studies have used internet search engine data (eg, Google Trends) to investigate temporal patterns in internet search activity on the keyword “insomnia.” In 2017, Ji and Kang [23] compared the search volume for keywords for 4 sleep disorders, including insomnia, during 22 months (January 2016-Octover 2017) using NAVER DataLab and Google Trends data. Other recent studies have focused on the longitudinal analysis of internet searches for insomnia during the 2019 COVID-19 worldwide pandemic. In 2020, Lin et al [24] reported the correlation between COVID-19–related deaths and the number of days with significantly increased searches for insomnia during the month after March 20, 2020, across 19 countries. In 2021, Zitting et al [25] also observed increased searches for insomnia during the first 5 months of the COVID-19 pandemic (January-May 2020) and its association with the cumulative number of COVID-19–related deaths worldwide and in the United States. In 2023, Lin et al [26] found increased internet searches for insomnia across 45 countries during the COVID-19 pandemic (March 2020-February 2021) and the mediation effect of stay-at-home levels on COVID-19 impacts on insomnia. These studies have demonstrated how internet search volume data can be used to reveal the longitudinal pattern in sleep concerns of the general population.

One of the major limitations of previous studies on the internet search volume is that different age groups cannot be identified in most cases (eg, search volume data from Google Trends). However, investigating the internet search activity of various age groups is feasible in NAVER DataLab, the most widely used internet search engine with the largest number of users in South Korea [27]. NAVER DataLab [28], which is a similar service to Google Trends, provides daily search volume data for various age groups, which are roughly divided by an age range of 5 years: ≤12 years, 13-18 years, 19-24 years, 25-29 years, …, 55-59 years, and ≥60 years. Such age information is available because NAVER, a multipurpose web portal service (similar to Yahoo but much more dominant in South Korea), has most users signed in with their accounts while using services like a web search. Due to Korean government regulations, NAVER has acquired domestic users’ personal information (name and date of birth) and verifies it with mobile phone user authentication. Therefore, NAVER DataLab can provide the daily search volume data for each age group in a nationwide population (with a certain level of anonymity), which creates a new opportunity for a comparison study across various age groups (eg, adolescents vs adults).

This study aimed to investigate the internet search pattern for the word “불면증” (*insomnia* in Korean) in the adolescent population (13-18 years old) compared to the adult population (19-59 years old) in South Korea. We hypothesized that adolescents might show distinct internet search patterns from adults, which might reflect the characteristic sleep problems in adolescents, especially concerning the school schedule (ie, academic vacations and weekends). Internet search activity data could be valuable to understanding distinctive temporal patterns in subjective sleep problems in a vulnerable age group, such as adolescents.

## Methods

### Recruitment

We used the NAVER DataLab search engine [28] to assess the trend in internet search activity in South Korean adolescent and adult populations. During the study period (from January 1, 2016, to December 31, 2019), the market share of NAVER DataLab among search engines in South Korea was 72.43% on average, which is overwhelmingly higher than that of other sites (Google comes second with 16.68%, and DAUM is third with 8.27%) [27]. NAVER provided the NAVER DataLab service, a publicly available web-based tool for investigating a temporal trend in web query activity on a daily, weekly, or monthly basis, from January 1, 2016. In this study, we examined the search query volumes for 불면증 (*insomnia* in Korean) for 4 years from January 1, 2016, to December 31, 2019. We focused on web search queries for insomnia only, similar to previous studies [24-26], as NAVER DataLab does not support the statistics for a search query with multiple words (ie, a search query including spaces, such like “I cannot fall asleep”). We set our study period up to December 31, 2019, so as to exclude the complicated influence of the COVID-19 pandemic on insomnia in the general population (we are pursuing this topic in another follow-up study).

We evaluated the daily search volume data for adolescents (13-18 years old) and adults (19-59 years old). We also extracted the daily search query data for young adults (19-24 years old) for additional comparison. NAVER DataLab provides the number of web queries (from each age group) on a relative scale from 0 to 100, where the maximal value in the given period is scaled to 100. The raw data are provided in Multimedia Appendix 1. In other words, the raw data from NAVER DataLab do not represent an absolute number of daily search queries. Instead, the search volume data are automatically scaled for each age group so that the maximum daily search volume in the given period (January 1, 2016-December 31, 2019, in this study) is 100, like in Google Trends. This original scale is highly variable with a single data point, the date with the peak search volume in the study period. In addition, this scaling is separately applied for each group, so the raw data cannot be directly compared across age groups. For group comparison, we normalized each group’s raw search volume data by the median value of each group for subsequent analysis. Our rationale was that the median of the daily search volume would be the robust representative value for the entire study period and relative fluctuations (above or below the median) could be more reliably comparable across groups. Within each group, we estimated the annual median value from raw search volume data and normalized the search volume data with the median value year by year. Next, we merged the normalized search volume data across 2016-2019 for later analysis. The final normalized search volume in our study represents whether the search volume for a given day is above (>1) or below (<1) the annual median in each age group.

We successfully separated slow fluctuation (over a year) and fast fluctuation (within a week) in the original trend using fast Fourier transform. The seasonal trend across the months in the year was examined in the slow fluctuation component (low pass–filtered data), and the weekly trend was analyzed using the fast fluctuation component (high pass–filtered data).

### Statistical Analysis

We conducted data analysis using MATLAB R2023a (MathWorks). We performed 2-way ANOVA to check for a significant group×month interaction in the seasonal trend and a group×day interaction in the weekly trend. After discovering a significant interaction in 2-way ANOVA, we performed group comparisons each month (or each day in a week) using the Wilcoxon rank sum test across age groups. We applied Bonferroni correction for multiple comparisons. In addition, the coefficient of variation, the ratio of the SD to the mean (σ/μ), was computed in each group to compare the relative magnitude of variation (across months in a year and days in a week, respectively).

### Ethical Considerations

We used publicly available anonymized information that can be obtained without special agreement. Data are publicly available and aggregated and thus no IRB approval or exemption is needed.

## Results

### Temporal Trend in Web Query Activity on Insomnia

**Figure 1:**
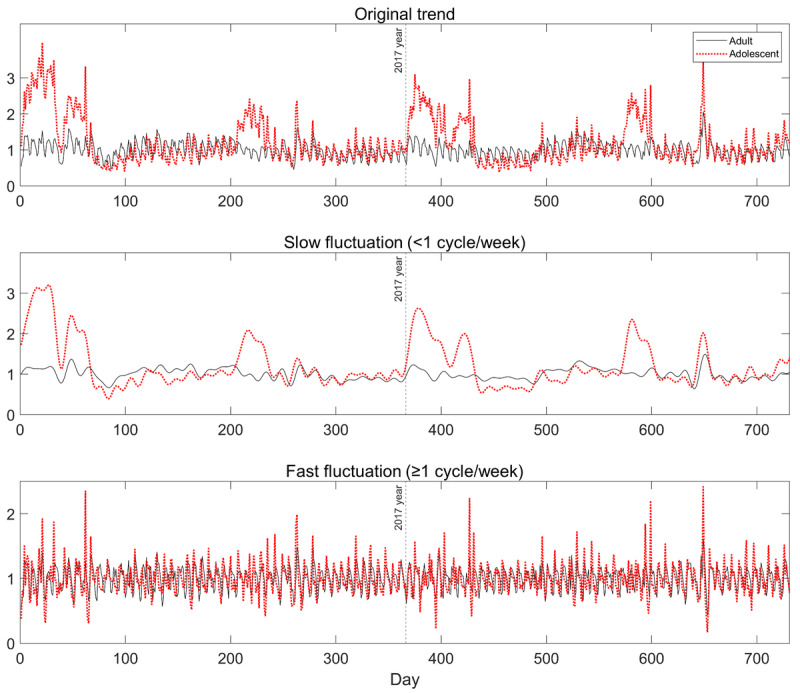
Slow and fast fluctuations in the search trend of insomnia in adults (19-59 years) and adolescents (13-18 years) during 2016-2017. The daily search volume was normalized within each group so that the average search volume over the entire period was 1. The original time series in each group (top) was decomposed into (middle) slow fluctuation (slower than 1 cycle per week) and (bottom) fast fluctuation (equal to or faster than 1 cycle per week) using fast Fourier transform. The search trend of young adults was omitted for simple visualization.

The temporal trend in web query activity on insomnia during 2016-2019 was examined in the adolescent (aged 13-18 years), adult (aged 19-59 years), and young adult (aged 19-24 years) populations in South Korea. Figure 1 (top panel) shows the original trend during 2016-2017 for visualization. We found that the original trend in the search volume was composed of a weekly pattern and a slower annual trend, as shown in Figure 1. The annual pattern remained similar in 2018 and 2019 as well. We separated slow fluctuation (across a year) and fast fluctuation (within a week) using fast Fourier transform, as shown in Figure 1 (middle and bottom panels).

#### Slow Fluctuation

**Figure 2:**
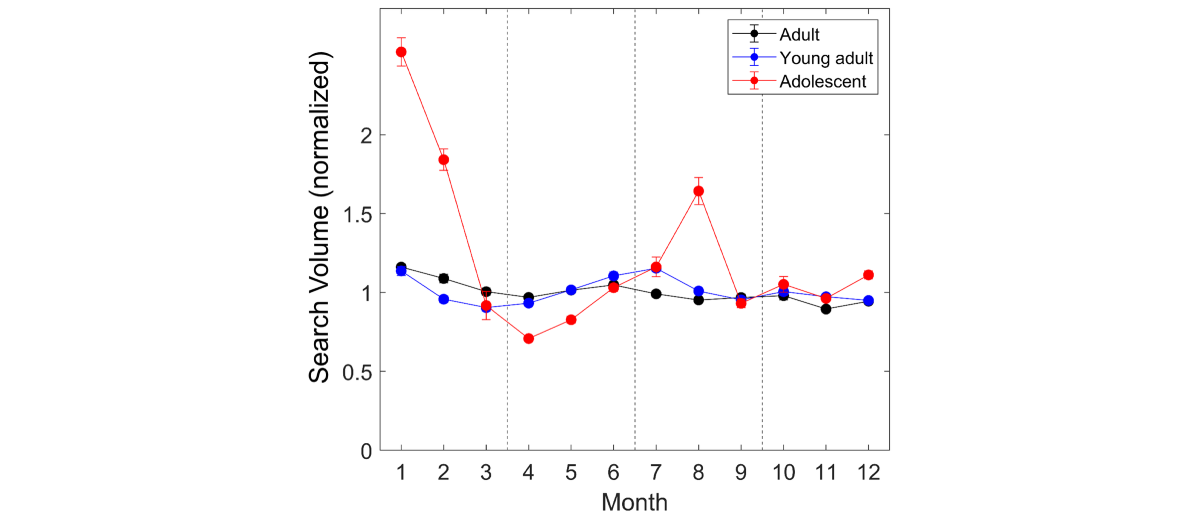
Comparison of seasonal trend in the search volume of insomnia across adults (19-59 years), young adults (19-24 years), and adolescents (13-18 years). Adolescents showed much greater seasonal fluctuation than adults and young adults (*P*<.001, group ×month interaction). Error bar: 95% CI. *P<.001 in independent t-tests with Bonferroni correction for group comparison.

We first analyzed each group’s annual trend in the slow fluctuation component. Korean adolescents (aged 13-18 years) demonstrated much greater seasonal fluctuation in the search volume than adults (aged 19-59 years) and young adults (aged 19-24 years), as shown in Figure 2. We found a significant group×month interaction in 2-way ANOVA (*F*_1,22_=230.1, *P*<.001). The normalized search volume for adolescents varied from 0.71 (April) to 2.52 (January) across a year, but that for adults varied between 0.89 (November) and 1.16 (January) only. The coefficient of variation was 0.483 and 0.131 for adolescents and adults, respectively. The adolescents’ search volume significantly increased in January, February, and August and relatively decreased in other months, except June, September, and October (*P*<.001, post hoc Tukey test.). In contrast, young adults exhibited a seasonal trend similar to adults (see Figure 2 and Table 1). The significant group difference between adolescents and adults in the annual trend was also observed between adolescents and young adults, except in November (all *P*<.005 after Bonferroni correction; see Table 1).

**Table 1:** Group comparison of the normalized daily search volume for insomnia across months^a^.

Month	Normalized daily search volume, mean (SD)				Corrected *P* value		
	Adolescents (13-18 years)	Adults (19-64 years)	Young adults (19-24 years)	Adolescents vs adults		Adolescents vs young adults	Young adults vs adults
January	2.52 (0.51)	1.16 (0.12)	1.14 (0.16)	<.001^b^		<.001^b^	>.99
February	1.84 (0.37)	1.09 (0.15)	0.96 (0.12)	<.001^b^		<.001^b^	<.001^b^
March	0.92 (0.51)	1.00 (0.13)	0.90 (0.14)	<.001^b^		<.001^b^	<.001^b^
April	0.71 (0.10)	0.97 (0.09)	0.93 (0.07)	<.001^b^		<.001^b^	<.001^b^
May	0.83 (0.12)	1.01 (0.10)	1.02 (0.11)	<.001^b^		<.001^b^	>.99
June	1.03 (0.13)	1.05 (0.12)	1.11 (0.14)	>.99		.011^b^	.041^b^
July	1.16 (0.35)	0.99 (0.12)	1.15 (0.12)	.003^b^		<.001^b^	<.001^b^
August	1.64 (0.49)	0.95 (0.08)	1.01 (0.09)	<.001^b^		<.001^b^	<.001^b^
September	0.93 (0.15)	0.97 (0.12)	0.95 (0.11)	.050		.291	>.99
October	1.05 (0.28)	0.98 (0.15)	1.00 (0.14)	>.99		>.99	.732
November	0.96 (0.10)	0.89 (0.07)	0.97 (0.05)	<.001^b^		>.99	<.001^b^
December	1.11 (0.14)	0.94 (0.07)	0.95 (0.08)	<.001^b^		<.001^b^	>.99

^a^Wilcoxon rank sum test with Bonferroni correction was conducted to compare significant group differences.

^b^Significant *P* values.

#### Fast Fluctuation

We also examined the weekly trend in the fast fluctuation of the search volume for each group, and all groups showed an obvious weekly pattern, as shown in Table 2. Two-way ANOVA revealed a significant group×day interaction (*F*_1,12_=66.4, *P*<.001). The coefficient of variation differed between adolescents and adults: 0.193 and 0.264, respectively. In all groups, the search volume for insomnia was the lowest on Saturday, with a decreasing trend during weekdays. In the group comparison, adolescents showed significantly increased search volumes on Monday, Sunday, and Saturday compared to adults but decreased search activity on other weekdays (ie, Tuesday-Friday; *P*<.001). See Table 2 for detailed statistics. The weekly pattern of search activity in young adults was similar to that for adults but distinct from that for adolescents (see Table 2). Although about 7 (70%) of 10 young adults in South Korea are undergraduate students [29], the observed seasonal and weekly trend in adolescents might be specific to teenagers, not undergraduate students. In summary, the adolescent population exhibited a relatively increased search volume for insomnia on Sunday and Monday than on other days.

**Table 2:** Group comparison of the normalized search volume for insomnia across days in a week^a^.

Day of the week	Normalized daily search volume, mean (SD)				Corrected *P* value		
	Adolescents (13-18 years)	Adults (19-64 years)	Young adults (19-24 years)	Adolescents vs adults		Adolescents vs young adults	Young adults vs adults
Monday	1.33 (0.28)	1.14 (0.12)	1.14 (0.12)	<.001^b^		<.001^b^	>.99
Tuesday	1.06 (0.20)	1.16 (0.11)	1.12 (0.12)	<.001^b^		<.001^b^	<.001^b^
Wednesday	1.01 (0.21)	1.13 (0.12)	1.11 (0.11)	<.001^b^		<.001^b^	.225
Thursday	0.97 (0.21)	1.08 (0.11)	1.05 (0.10)	<.001^b^		<.001^b^	.126
Friday	0.85 (0.18)	0.97 (0.11)	0.95 (0.10)	<.001^b^		<.001^b^	.338
Saturday	0.78 (0.17)	0.75 (0.08)	0.81 (0.10)	.003^b^		>.99	<.001^b^
Sunday	1.01 (0.19)	0.78 (0.11)	0.83 (0.08)	<.001^b^		<.001^b^	<.001^b^

^a^Wilcoxon rank sum test with Bonferroni correction was conducted to compare significant group differences.

^b^Significant *P* values.

**Figure 3:**
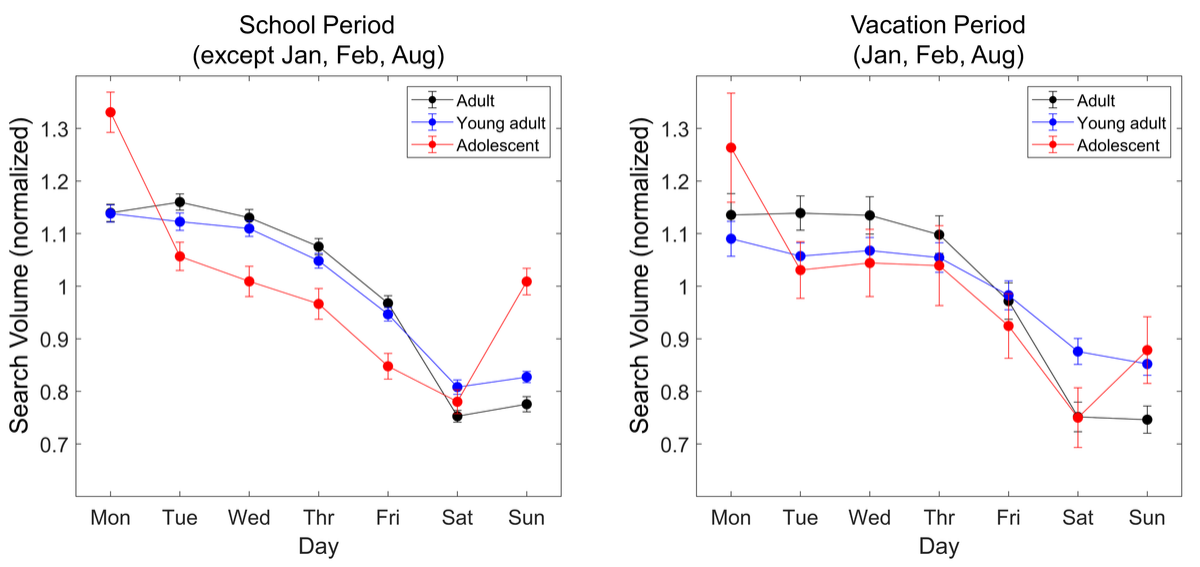
Comparison of the weekly trend in the search volume for insomniaacross adults (19-59 years), young adults (19-24 years), and adolescents (13-18 years): (left) school period (all months except January, February, and August) and (right) vacation period (January, February, and August). Error bar: 95% CI.

In addition, we compared the weekly pattern of search activity in adolescents between the vacation months (January, February, and August) and the remaining months (see Figure 3), as the slow seasonal trend in adolescents’ search activity was primarily affected by academic vacations in a year. In adolescents, an increased search volume for insomnia on Sunday and Monday was also observed during academic vacations (January, February, and August) but in a diminished magnitude compared to the school period.

## Discussion

### Principal Findings

This study analyzed the time series of internet search activity in the popular search engine platform in South Korea (ie, NAVER DataLab) to discover the daily search trend for insomnia in South Korean populations, particularly comparing adolescents versus adults. The adolescents demonstrated significantly increased search activity on insomnia in January, February, and August across a year and on Sunday and Monday in a week. These distinctive seasonal and weekly trends in adolescents suggest their characteristic sleep problems, particularly concerning the school schedule. Therefore, it is favorable to consider these temporal characteristics in adolescents’ sleep problems and the biological and social factors underlying them.

The seasonal pattern (across a year) of the search activity in adolescents seemed to be strongly coupled with academic vacations in South Korea: Middle and high schools in South Korea usually have summer vacation from the end of July to the end of August and winter vacation between the end of December and the end of February. A potential explanation is that adolescents’ subjective sleep disturbance is more affected by late sleep onset (and the delayed sleep phase) than the absolute amount of sleep. Delayed bedtime and increased sleep onset latency during vacations have been frequently observed in many studies tracking sleep patterns of adolescents [30-34]. Hansen et al [32] reported delayed bedtime and increased total sleep time during vacations and weekends compared to school nights. In a study with 127 high school students in Brazil, both bedtime and rise time were substantially delayed during vacations (107 minutes and 208 minutes on average, respectively) compared to school days [34]. A recent study using actigraphy also reported considerable delays in bedtime (from 23:17 to 00:38 hours) and rise time (from 07:20 to 09:37 hours) during vacation weeks [30]. In this study, sleep onset latency gradually increased during the 2 weeks of the vacation, suggesting that the delayed sleep schedule during vacations might induce difficulty initiating sleep. A consequent study also reported that online social media activity in the evening is associated with presleep arousal and late sleep onset during vacations [33]. Recently, it has been suggested that adolescent insomnia might be related to poor sleep hygiene and the delayed sleep phase [34]. Because of unconstrained sleep opportunities during vacations, adolescents would sleep any time they want, eventually resulting in delayed sleep. Poor sleep hygiene also affects sleep negatively at this time. Eventually, adolescents might struggle to initiate sleep due to inadequate sleep hygiene and the delayed sleep phase, even though the total sleep time increases during vacations. Further study is required to confirm whether adolescents’ insomnia symptoms during vacations are primarily characterized by sleep initiation difficulty with the delayed sleep phase.

In contrast, the similar age group of the early twenties (19-24 years) did not demonstrate such profoundly increased search activity on insomnia during vacation months. It implies that this subjective sleep problem during vacations is rather selectively observed in the adolescent population. The disrupted daily routines during their summer or winter vacation may be shared among many college students (most of the young adult group in South Korea). The enormous seasonal variation of sleep concerns in adolescents might result from a synergistic effect between the disrupted sleep routine and the physiological factors in the adolescent population, as adolescents have a progressive circadian delay and a reduced homeostatic sleep drive [10,11].

The weekly pattern of search activity implies that adolescents might suffer from increased insomnia symptoms from Sunday to Monday. The increased search volume on Monday might reflect search queries right after midnight (eg, midnight-3:00 A.M. on Monday), as the average weekend bedtime in adolescents is around 12:14 A.M. (SD 1 hour 17 minutes) in South Korea [14]. Delayed bedtime on weekend nights is commonly observed in other countries as well [5,7].

Several potential reasons may explain increased sleep concerns on Sunday and Monday in adolescents. First, they might have difficulty changing lifestyle patterns from weekends to weekdays and suffer excessive stress in waking up early for school. Previous studies have reported that adolescents’ sleep phase is relatively delayed [35,36]. On weekdays, adolescents have to wake up early for school and usually suffer from insufficient sleep. It is a worldwide phenomenon that adolescents lack sleep time as their bedtime is delayed, but the time for school stays the same [37]. However, they tend to sleep late during the weekend, as they are free from the pressure of a fixed wakeup time. Adolescents are more exposed to stimulating activities, such as social media, video games, and watching movies late at night and on weekends [38]. Therefore, adolescents may have searched for insomnia a lot because they had difficulty falling asleep at night due to a delay in the sleep phase rather than insomnia disorder (in the clinical definition). In addition, adolescents usually sleep much longer during the weekend (catch-up sleep) to recover from sleep deprivation during weekdays [5,7,14,39]. Increased sleep time involves a much greater rise time delay (compared to bedtime delay), which exaggerates the delayed sleep phase during the weekend.

Previous adolescent studies have consistently reported increased sleep duration (catch-up sleep) and delayed sleep patterns during weekends [5,7,14,39]. According to a meta-analysis by Gradisar et al [5], bedtime was delayed by 122.3 minutes, and the total sleep time increased by 91.6 minutes when comparing weekend and weekday sleep behaviors. A recent study on South Korean adolescents also observed a delayed sleep pattern (bedtime delay 49.5 minutes, rise time delay 163.8 minutes) and increased total sleep time (127.5 minutes on average) on weekends [39]. Eventually, adolescents’ sleep behavior during weekends deviated substantially from weekdays in the sleep phase and the total sleep duration. Adolescents are forced to adapt to markedly different sleep patterns on Sunday night. They need to bring forward the sleep phase to match the school time and reduce the total sleep time, which can be a tremendous stress for the circadian rhythm. In a recent study, 2 weeks of therapeutic intervention (evening routines, gradual bedtime adjustment, and bright-light exposure) successfully resulted in increased sleep duration, earlier sleep onset, improvement in sleepiness and moods, and adjustment of the circadian rhythm [40]. This finding supports the idea that adjusting the circadian rhythm and sleep phase might be essential in improving adolescent sleep.

Another possibility is that adolescents might have psychological stress from going to school. The worries and stresses are more intense on Sunday nights when they have to get ready for school again. South Korean students are in a competitive environment to achieve better grades and are forced to study intensively for high-ranking universities [41]. In addition, school bullying is not uncommon, and previous studies have shown that 6.4%-14.1% of South Korean adolescents experience bullying victimization [42-44]. Given that the stress from academic performance or bullying has been a social problem, it is not difficult to predict that these adolescents will not be able to sleep well due to worries about their next 5-day school life. Teenagers with a shorter sleep duration feel much stressed and have more suicidal ideation [8]. This could be related to the suicide rate of South Korean teenagers, which is remarkably higher on “Blue Monday” compared to other ages [45].

South Korean adults also have an average sleep time of 419.44 minutes on weekdays and tend to catch up on their sleep on weekends. Even the term “Monday sickness” is widely used in adults because they hate going to work on Mondays [46]. However, the search trend for insomnia in adults did not specifically increase on Monday in comparison to other weekdays (eg, Tuesday). This suggests that adolescents get stressed with weekday daily life more severely than adults due to physiological and social factors.

### Limitations

This study has a few limitations. First, the search term “insomnia” could encompass a wide range of sleep-related concerns, from clinically diagnosable insomnia to more general difficulties with sleep or perceived insufficient sleep. In this study, we did not define insomnia based on specific symptoms or duration. Users searching for this term might be experiencing various sleep-related issues, not necessarily meeting the clinical criteria for insomnia.

Second, we assumed that the internet search queries on insomnia might reflect seeking information about subjectively experienced sleep difficulty, but there are other possibilities. For example, adolescents may survey insomnia for academic purposes or look for something else under the title of insomnia (eg, music bands, songs, movies). We cannot confirm what fraction of the total search queries is irrelevant to medical needs. Still, we observed profound weekly and seasonal patterns in the adolescents’ internet search activity, and these are more likely to reflect subjective sleep concerns that are associated with the school schedule.

Third, we acquired the daily search volume of each age group on a relative scale only, not as an absolute number. The finding that adolescents exhibit a higher normalized search volume on Monday than adults does not necessarily mean that adolescents ran a larger number of search queries than adults. Instead, it indicates that search queries were relatively more concentrated on Monday for adolescents than for adults (or more largely deviated from the group median).

Finally, the results may reflect the specific properties of South Korean youths because it is a search engine mainly used by South Korean people. South Korean students have the highest level of academic stress among the Organization for Economic Co-operation and Development (OECD) countries [47] and have fewer sleeping hours than other countries generally [5,48]. However, sleep deprivation in adolescents is a worldwide problem, and many studies have been conducted to determine teenagers’ sleep patterns in many countries [5,49]. This study is meaningful for confirming the trend among adolescents in using the internet, one of the most accessible sources of information in everyday life.

Future research may compare the trends in search engines in other countries, including Google. More direct data about the characteristics of sleep problems could be obtained by the smartphone’s sleep log apps (on a relatively large scale). In addition, it would be meaningful to analyze medical insurance data to check whether internet search activity leads to actual visits to sleep clinics (eg, aggregating anonymized data from different periods or regions).

### Comparison With Prior Work

Big data analysis based on the internet search engine platform has advantages, such as cost-effectiveness and relative accuracy based on real-world human behaviors [50,51]. Carneiro et al [52] analyzed Google web search data on influenza to predict the expansion of influenza in the United States 7-10 days before the Centers for Disease Control and Prevention. After Carneiro et al’s [52] work, many infodemiology and infoveillance studies have used big data based on internal search engines or social media. These studies on internet search trends have focused on predicting disease prevalence to prevent public health problems [53].

Several recent studies have also investigated the longitudinal pattern of internet searches for insomnia among the general population [23-26]. Most studies have focused on increased internet searches for insomnia during the early COVID-19 pandemic. Investigations on the internet search activity before the COVID-19 pandemic are relatively scarce. Ji and Kang [23] compared the relative search volume across 4 types of sleep disorders (insomnia, sleep apnea, snoring, and restless leg syndrome) and examined an overall increasing or decreasing trend over 22 months. Zitting et al [25] observed the weekly fluctuation during the prepandemic period (2017-2019), during which the internet search volume was the largest on Sundays and Mondays and decreased over weekdays. In addition, weekly patterns significantly altered during the first 20 weeks of 2020, the early COVID-19 pandemic. However, these studies have neither separated fast fluctuation in a week and slow fluctuation in a year nor specifically assessed the adolescent group.

In this study, we enabled the comparison between different age groups (ie, adolescents vs adults) using NAVAR DataLab. As noted in the *Introduction* section, NAVER DataLab, which compares the daily internet search activity in various age groups, can open up a new opportunity for infodemiology research. This is a great advantage compared to the data from other search engines (eg, Google Trends). This study can be an initial example of using NAVER DataLab to compare various age groups in infodemiology and infoveillance studies.

Traditionally, most epidemiologic studies of sleep disorders among adolescents use sleep diaries or sleep questionnaires. O’Sullivan et al [27] found no significant differences in measurements when using Actigraphy and sleep diaries for sleep parameter estimates. Conventional methods, such as sleep diaries and actigraphy, can provide detailed information about the individual’s sleep behavior pattern, but it is not easy to apply them in a large population (eg, more than thousands of participants) or for a long period. In contrast, our approach using internet search query data can easily estimate the temporal trend along an extended period (eg, an entire year or longer) at the whole population level. Thus, internet search volume data are a complementary information source that can be combined with the findings in conventional studies for further validation. For example, Blunden et al [31] showed a clear weekly pattern in the bedtime and rise time of adolescents during a school term (but not evident in the total sleep time and sleep efficiency). Taken together, we can hypothesize that delayed bedtime and rise time (ie, delayed sleep phase) are more likely to contribute to the increased search volume on Sunday and Monday in this study. Combining these distinct types of information sources will be helpful in validating, interpreting, and generalizing the results from both sides.

### Conclusion

In conclusion, this study demonstrated that adolescents have a distinct tendency to search the internet for insomnia compared to adults. In adolescents, much more searches for insomnia are observed on Sunday, Monday, and academic vacations in summer and winter. These results reflect the characteristics of sleep problems in adolescents, suggesting that their sleep concerns are more from adjustment between substantially different sleep patterns between school days and nonschool days (ie, vacations and weekends). These results align with previous studies using questionnaires and actigraphy [19]. We hope our results will be used in developing policies and treatment plans for adolescents’ sleep problems to improve the quality and quantity of sleep.

## Appendix

Multimedia Appendix 1 Raw data from NAVER DataLab.
